# Global Decrease of Histone H3K27 Acetylation in ZEB1-Induced Epithelial to Mesenchymal Transition in Lung Cancer Cells

**DOI:** 10.3390/cancers5020334

**Published:** 2013-04-03

**Authors:** Joëlle Roche, Patrick Nasarre, Robert Gemmill, Aleksander Baldys, Julien Pontis, Christopher Korch, Joëlle Guilhot, Slimane Ait-Si-Ali, Harry Drabkin

**Affiliations:** 1 Department of Medicine, Hematology Oncology Division, MUSC, 96 Jonathan Lucas St., Charleston, SC 29425, USA; E-Mails: nasarre@musc.edu (P.N.); gemmill@musc.edu (R.G.); drabkin@musc.edu (H.D.); 2 CNRS FRE 3511, University of Poitiers, 1 rue Georges Bonnet, F-86022 Poitiers Cédex, France; 3 Department of Medicine, Nephrology Division, MUSC, Ralph H. Johnson Veterans Affairs Medical Center, Charleston, SC 29425, USA; E-Mail: aleksander.baldys@siemens.com; 4 Epigénétique & Destin Cellulaire, CNRS UMR 7216, University of Paris Diderot, Sorbonne Paris Cité, F-75013 Paris, France; E-Mails: julien.pontis@univ-paris-diderot.fr (J.P.); slimane.aitsiali@univ-paris-diderot.fr (S.A.); 5 CU DNA Sequencing and Analysis Core, University of Colorado, School of Medicine, Anschutz Medical Campus, 12801 E. 17th Ave., Aurora, CO 80045, USA; E-Mail: Christopher.Korch@ucdenver.edu; 6 INSERM, CIC 0802, CHU de Poitiers, F-86021 France; E-Mail: joelle.guilhot-gaudeffroy@chu-poitiers.fr

**Keywords:** EMT, ZEB1, lung cancer, histone acetylation, RAB25

## Abstract

The epithelial to mesenchymal transition (EMT) enables epithelial cells with a migratory mesenchymal phenotype. It is activated in cancer cells and is involved in invasion, metastasis and stem-like properties. ZEB1, an E-box binding transcription factor, is a major suppressor of epithelial genes in lung cancer. In the present study, we show that in H358 non-small cell lung cancer cells, ZEB1 downregulates *EpCAM* (coding for an epithelial cell adhesion molecule), *ESRP1* (epithelial splicing regulatory protein), *ST14* (a membrane associated serine protease involved in HGF processing) and *RAB25* (a small G-protein) by direct binding to these genes. Following ZEB1 induction, acetylation of histone H4 and histone H3 on lysine 9 (H3K9) and 27 (H3K27) was decreased on ZEB1 binding sites on these genes as demonstrated by chromatin immunoprecipitation. Of note, decreased H3K27 acetylation could be also detected by western blot and immunocytochemistry in ZEB1 induced cells. In lung cancers, H3K27 acetylation level was higher in the tumor compartment than in the corresponding stroma where ZEB1 was more often expressed. Since HDAC and DNA methylation inhibitors increased expression of ZEB1 target genes, targeting these epigenetic modifications would be expected to reduce metastasis.

## 1. Introduction

The epithelial-to-mesenchymal transition (EMT), which converts epithelial cells into an elongated, motile and invasive phenotype, is thought to be a critical step in the dissemination of tumor cells during metastasis [1,2]. For example, the loss of E-cadherin and upregulation of vimentin or *N*-cadherin have been most frequently described during EMT. Other frequent changes include the loss of cytokeratins, increased MMP activity, and increased fibronectin. In epithelial cancers, EMT has been associated with resistance to therapy and a poor prognosis [3,4]. In part, EMT is mediated by transcription factors including ZEB1, ZEB2, Snail, Slug, Twist and E12/E47 that bind E-box elements (*i.e.*, CANNTG) in genomic DNA [5,6]. In lung cancer, ZEB1 appears to be a major factor in the EMT process [7]. In non-small cell lung cancer (NSCLC) cell lines, we and others previously found that loss of E-cadherin was inversely and specifically correlated with ZEB1 mRNA expression [7,8]. In addition, we reported that the tumor suppressor gene *SEMA3F*, coding for a cell guidance and tumor suppressor molecule, was directly repressed by ZEB1 in H358 NSCLC cells [9]. 

By transcriptomic analyses of 38 NSCLC cell lines, we identified 466 genes that were significantly correlated with ZEB1 expression [10]. For a subset of genes, the response to ZEB1, ZEB2, and TGFβ (a natural inducer of EMT) was confirmed. These genes include *ST14*, encoding for a membrane associated serine protease (matriptase) that processes the hepatocyte growth factor precursor and plays a role in tight-junction maintenance; *EpCAM*, encoding for an epithelial cell adhesion protein targeted by the therapeutic antibody catamaxumab; and *ESRP1*, encoding an epithelial splicing factor, which plays an important role in EMT biologic responses [10]. However, we did not determine whether ZEB1 binds directly to these genes. Also included in the gene set, but not further explored, was *RAB25* encoding an epithelial-specific member of the Rab family of small GTPases, which can act as both a tumor enhancer and suppressor depending on the cellular context. RAB25 is involved in cell migration, invasion, and intracellular vesicle trafficking in the regulation of epithelial polarity and transformation [11,12,13]. This may include the recycling of EGF and TGF-β receptors [14]. In addition, the reported binding of RAB25 to Smad4 and TGFβR1 suggests that its regulation by ZEB1 may have an important role in TGF-β signaling [15,16].

ZEB1 protein level is negatively regulated by the miR-200 microRNA family, which includes five members (miR-200a, -b, -c, -141, and -429). A positive feedback loop has been described with ZEB1 being able to repress miR-200c and miR-141 expression (for a review see [17]). The transcriptional activity of ZEB1 is mediated through recruitment of co-repressors and co-activators depending on the target gene and tissue [18]. This complexity underlies the multiple functions of ZEB1 as a transcriptional activator as well as repressor. For gene activation, ZEB1 associates with the histone acetyltransferases (HATs) p300, PCAF, and Tip60. In contrast, as a repressor, ZEB1 interacts with CtBP [19] and recruits class I and II histone deacetylases (HDACs) in pancreatic tumors to repress the E-cadherin gene *CDH1* [20]. ZEB1 can also recruit the nicotinamide adenine dinucleotide-dependent HDAC SIRT1 in prostate cancer cells to repress *E-cadherin* and to induce several EMT markers [21]. Partners like repressive histone lysine methyltransferases (KMTs) and the lysine demethylase (KDM) LSD1 bind ZEB1 (for reviews see [22,23]). In addition, ZEB1 interacts via its *N*-terminal region with BRG1 to repress *E-cadherin* in colon cancer cells [24]. BRG1 is one of the two ATPase subunits in the SWI/SNF chromatin-remodeling complex and has been reported to be frequently mutated or silenced in primary human NSCLC tumors and cell lines (for reviews see [25,26]). Therefore, we would anticipate that the induction of ZEB1 would lead to epigenetic changes shown to be important in cancer causation and progression [27].

In the present study, we tested the hypothesis that ZEB1 directly binds *ST14*, *EpCAM*, and *ESRP1* genes in H358 non-small cell lung cancer cells. We also validated *RAB25* as a ZEB1 target gene. For each of these genes and other ZEB1 targets, we asked whether ZEB1 binding induced changes in histone marks (acetylation and methylation). We found direct ZEB1 binding to target genes associated with decreased histone acetylation. Lastly, human lung tumors were tested for ZEB1 and H3K27 acetylation by immunohistochemical staining. In these tumors, H3K27 acetylation level was higher in the tumor compartment than in the corresponding stroma where ZEB1 is more often expressed.

## 2. Experimental Section

### 2.1. Cell Lines and Transformants

NSCLC cell lines were obtained from the Colorado Lung Cancer SPORE Cell Repository. Verification of cell lines was carried out by microsatellite genotyping analysis and comparison to ATCC data. Cell lines were grown in RPMI-1640 supplemented with 10% FCS and antibiotics (Invitrogen, Carlsbad, CA, USA) at 37 °C and 5% CO_2_. Non-immortalized (NHBE), telomerase-immortalized (FC6625-2 3KT) and SV40-immortalized (BEAS2B) human broncho-epithelial cells were grown as described [10].

NCI-H358 cells (hereafter H358) containing an empty vector (EV) or doxycycline-inducible myc-tagged *ZEB1* have been described [10]; these were grown with 100 µg/mL hygromycin and 5 µg/mL blasticidin (Invitrogen) for selection. ZEB1 was induced by 100 ng/mL doxycycline (DOX) (Invitrogen) for 48 h. TGF-β treatment was for 48 h with 10 ng/mL [10]. Treatment with 5 µM 5-aza-2'-deoxycytidine (AZA, Sigma, Saint Louis, MO, USA) or 5 µM vorinostat (SAHA, Merck, Rahway, NJ, USA) was performed for 48 h and 16 h, respectively. When both were used in combination, SAHA was added during the last 16 h of treatment with AZA. The medium was changed after 24 h and fresh AZA solution prepared each time from a stock solution in 100% DMSO.

### 2.2. RNA Expression Analysis

Total RNA was extracted using the RNeasy Mini kit (Qiagen, Valencia, CA, USA) with DNase I treatment and quality controlled by electrophoresis on 0.8% agarose gel. RT-PCR was performed on 0.5–1 μg total RNA with the Transcription First Strand cDNA Synthesis kit from Roche (Mannheim, Germany). Gene expression was assessed by quantitative real-time PCR with the GeneAmp 7500 system and SYBR-Green chemistry (Applied Biosystems, Foster City, CA, USA). Data were expressed as the percent of GAPDH, 100 × 2^−ΔCt^, where ΔCt = Ct_gene of interest_ − Ct_GAPDH_. Primer sequences are provided in Table 1.

**Table 1:** Primers for qPCR.

Gene	Forward Primer (5' to 3')	Reverse Primer (5' to 3')
**Primers for qRT-PCR**		
Actin	ATGACTTCCAAGCTGGCCG	CCTTGGCAAAACTGCACCTTC
EpCAM	CGCAGCTCAGGAAGAATGTG	TGAAGTACACTGGCATTGACGA
ESRP1	TCCTGCTGTTCTGGAAAGTCG	TCCGGTCTAACTAGCACTTCGTG
CDH1 *	CGGGAATGCAGTTGAGGATC	AGGATGGTGTAAGCGATGGC
GAPDH	TGCACCACCAACTGCTTAGC	GGCATGGACTGTGGTCATGAG
NRP2	GGATGGCATTCCACATGTTG	ACCAGGTAGTAACGCGCAGAG
RAB25	AATGTTCGCTGAAAACAATGGAC	CTCAAAGGCTAGCTCAACATTGG
SEMA3F	AGCAGACCCAGGACGTGAG	AAGACCATGCGAATATCAGCC
ST14	GGGACACACCCAGTATGGAGG	GAGGTTCTCGCAGGTGGTCTG
ZEB1	AGCAGTGAAAGAGAAGGGAATGC	GGTCCTCTTCAGGTGCCTCAG
**Primers for ChIP qPCR**		
Alu	GCCTGT AATCCCAGCACTTT	AAGCGATTCTCCTGCCTCAGC
EpCAM	TAGCCTCCACGTTCCTCTATCC	TGCTGAGACTTCCTTTTAACCG
ESRP1	TCAGTCCTCCGCAACTTAGCT	TCCGAGACCCCACCTCGT
CDH1	GGCCGGCAGGTGAACCCTCA	GGGCTGGAGTCTGAACTGA
NRP2	TTAACCCACCCTGGAGTCTCC	GCAATAGCTGCTAATTTGAGCG
RAB25	CACCCAACCTGTCGAACCT	GAGAGGACGGAAGCTGAGAAC
SEMA3F-pr	GGCGTATGGATGTGTGGATGA	TATGAGAGCACCCACCCAGAAC
SEMA3F-neg	CCCTACAGTTCCAGCAGCCC	CCACCAACCCAGACCCTGAT
ST14-pr	CAAAGTGAGCAAGGTGAAGGG	TTTATCCACCTCCTTGATGCC
ST14-neg	ATCTCCCACCCCTTCTTCAATG	GCTGTACTCTGCCGGTTTCTC

CDH1 *: codes for E-cadherin.

### 2.3. MiRNA Analysis

Total RNA was extracted by TRIzol (Invitrogen). Reverse transcription for mature miR-200c and RNU6B was performed with 20 ng total RNA with the TaqMan MicroRNA reverse transcription kit containing MuLV reverse transcriptase (Applied Biosystems). The corresponding TaqMan MicroRNA assay was used for quantitative real-time PCR (Applied Biosystems). Results were reported as the percent of RNU6B with the ∆Ct method as above.

### 2.4. Chromatin Immunoprecipitation (ChIP) Assay

ChIP assay was performed with the protocol described by Upstate (Millipore, Saint Quentin en Yvelines, France) with modifications. Briefly, cells (10^7^ cells for ZEB1 ChIP and 10^6^ cells for histone modifications per assay) were cross-linked with 1% formaldehyde for 10 min at room temperature. Cells were resuspended in SDS lysis buffer [1% SDS, 10 mM EDTA, 50 mM Tris-HCl (pH 8.1)] for 10 min on ice. The lysate was sonicated with a Bioruptor Sonicator (Diagenode Inc, Sparta, NJ, USA) three times for 7 min each with water change. After centrifugation at 13,000 rpm at 4 °C for 10 min, the supernatant was diluted 10-fold in dilution buffer [0.01% SDS, 1.1% Triton ×100, 1.2 mM EDTA, 167 mM NaCl, 16.7 mM Tris-HCl (pH 8.1)] and incubated for 1.5 h on a rotating plateform at 4 °C with Protein-A Sepharose (Millipore, Temecula, CA, USA). Rabbit anti-ZEB-1 antibodies and rabbit polyclonal antibodies for histone modifications, were incubated with the pre-cleared chromatin on a rotating plateform overnight at 4 °C (Table 2). IgG from a non-immunized rabbit was used as a control. Immune complexes were collected with Protein-A Sepharose and washed 3 times with LiCl buffer [0.25 M LiCl, 1% Triton ×100, 1% deoxycholic acid, 1 mM EDTA, 10 mM Tris-HCl (pH 8.1)], then twice with TE buffer [1 mM EDTA, 10 mM Tris-HCl (pH 8)] before phenol-chloroform extraction and ethanol precipitation. Quantitative real-time PCR was performed with SYBR-Green chemistry with primers described in Table 1. The primers were designed to amplify evolutionary conserved regions with predicted ZEB1 binding sites as determined with ecrbrowser as published previously [9]. For each PCR primer set, Ct values were obtained for purified input DNA and the immunoprecipitated chromatin. The results were expressed as the percentage of the input: 100 × 2^−ΔCt^ where ΔCt_tested antibody_ = Ct_tested antibody_ − Ct_input_.

**Table 2:** Antibodies for ChIP, western blot, immunocytochemistry, and immunohistochemistry. References, quantities and working dilutions are indicated. IF: immunofluorescence, WB: western blot, TMA: tissue microarray.

Primary antibodies	Reference	Lot	ChIP	IF	WB	TMA
Rabbit control serum	Sigma I5006		10 µg			
Rabbit anti-ZEB1	Santa Cruz Biotechnology, H102	D2010	10 µg	1:50		1:50
Mouse monoclonal anti-ZEB1	R&D MAB 6708	CEZB0111021		1:50		
Rabbit anti-acetyl histone H3K9	Active Motive 39137	01008001	10 µL			
Rabbit anti-acetyl histone H3K27	Active Motive 39133	3161003	10 µg	1:500	1:20,000	1:50
Rabbit anti-acetyl histone H3	Millipore 06-599	DAM1823380	10 µg	1:500	1:20,000	1:50
Rabbit anti-acetyl histone H4	Millipore 06-866	JBC1873473	10 µg		1:20,000	
Rabbit monoclonal anti-E-cadherin	Cell Signaling 24E10	2		1:100	1:1,000	
Mouse anti-E-cadherin	B&D 610181	85521		1:100		
Anti-Rab25	Sigma R8532				1:1,000	
Anti-actin	Sigma				1:50,000	

### 2.5. Protein Detection by Immunoblotting

Immunoblots were performed as described [10]. For histone modifications, cells were directly lysed in Laemmli loading buffer and sonicated. Proteins were heat-denatured before electrophoresis on 15% polyacrylamide gels (BioRad, Hercules, CA, USA). Rabbit primary antibodies included anti-histone H3, anti-acetyl histone H3, anti-acetyl H3K27, and anti-acetyl histone H4 antibodies (Table 2). HRP-conjugated anti-rabbit secondary antibodies (1:5,000) were from Bio-Rad. Detection was performed with Western Lightning Plus ECL reagent (PerkinElmer, Waltham, MA, USA). 

### 2.6. Immunofluorescence

Cells grown on 8-wells ibidi plates (ibidi GmbH, Martinsried, Germany) were fixed for 15 min with 3.7% formaldehyde, permeabilized with 0.5% Triton X-100, and blocked using 3% goat serum in PBS. Rabbit anti-human ZEB1, mouse anti-human E-cadherin, rabbit anti-acetyl H3, anti-acetyl H3K27, and anti-acetyl H4 antibodies (Table 2) were incubated overnight at 4 °C. Mouse anti-human ZEB1 and rabbit anti-human E-cadherin antibodies were also tested. After three washes with PBS, cells were incubated for one hour with Alexa^488^-conjugated chick anti-mouse or Alexa^549^-conjugated goat anti-rabbit secondary antibodies. DAPI staining was performed for ten minutes. Stained slides were mounted in Dako Fluorescent Mounting Medium (Dako, Glostrup, Denmark). Images were captured with IPLab software on a BD CARVII spinning disc confocal microscope (BD Biosciences, San Jose, CA, USA).

### 2.7. Lung Cancer Tissue Microarray (TMA), Immuno-Histochemistry and Analysis

Commercial TMA slides containing 109 lung cancers and 10 normal lung tissues with evaluation of TNM disease stages were obtained from U.S. Biomax (Rockville, MD, USA, catalog reference BC041115). The detailed immunohistochemistry protocol has been described previously [10]. Briefly, slides were deparaffinized by incubations in Histoclear solution (National Diagnostics, Inc., Charlotte, NC, USA), rehydrated by sequential immersions in decreasing concentrations of ethanol and successively incubated in the following buffers: Antigen Unmasking Solution (Vector Laboratories, Burlingame, CA, USA), 0.3% H_2_O_2_, 0.5% Triton in PBS, and 5% rabbit serum in PBS with 0.1% Triton (Invitrogen). Rabbit primary antibodies were anti-human ZEB1, anti-acetyl histone H3 and anti-acetyl H3K27 (Table 2). Secondary antibodies (Vector Laboratories) were biotinylated goat anti-rabbit antibodies. Slides were exposed to Vectastain ABC standard solution (Vector Laboratories), then DAB peroxidase substrate (ThermoFisher Scientific, Inc., Waltham, MA, USA), and finally mounted using Dako Fluorescent mounting medium (Dako, Carpinteria, CA, USA). Slides were scanned with an Aperio system (Aperio Technologies, Inc., Vista, CA, USA) and each sample scored (from 0 to 300) by multiplying the percentage of positive cells in the tumor or stroma (0 to 100%) by the average level of staining intensity (0 to 3).

### 2.8. Statistical Analysis

Data were summarized by median and range for quantitative variables, percentage and confidence intervals when appropriate for qualitative variables. Expression score distributions in tumor, stroma and normal tissue were compared using Wilcoxon-Rank sum test and the Kruskall-Wallis test. Relationship between co-staining status and qualitative parameters (histology, nodes) was analyzed by Fisher’s exact test or Chi-square as appropriate. Correlations between score expression and other quantitative parameters were determined with the Spearman rank correlation method. Potential relationships with baseline characteristics were also explored with the use of non-parametric test, as appropriate.

## 3. Results

### 3.1. ZEB1 Binding to New Target Gene Promoter Regions

**Figure 1:**
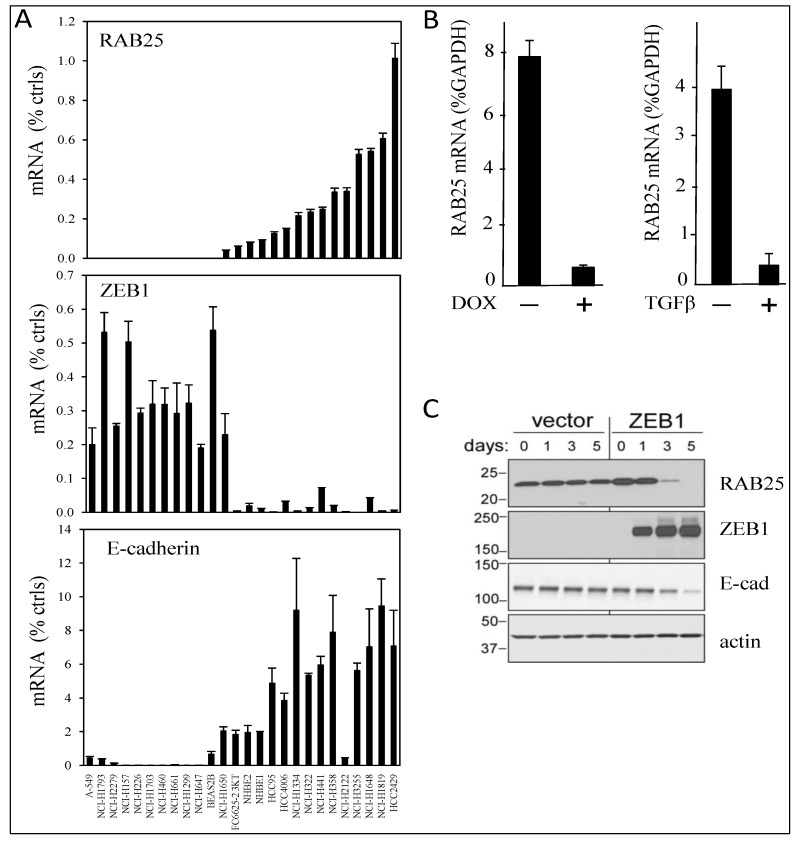
RAB25 is a ZEB1 target gene. (**A**) RAB25 expression positively correlated with E-cadherin but negatively with ZEB1 in a series of lung cancer cell lines. RNA expression was measured by quantitative real-time PCR in 22 NSCLC cell lines, two NHBE cultures, and two immortalized human airway primary cell lines (BAES2B and FC6625-2 3KT). Cells are ranked from left to right with increasing RAB25 mRNA level. Values are expressed as percent of the geometric mean between GAPDH and actin mRNA. The experiment was done twice with qRT-PCR in duplicate. (**B**) RAB25 mRNA level is decreased by ZEB1 overexpression in H358 FlipIn ZEB1 cells (left) and by TGFβ treatment in H358 EV control cells (right). Values are expressed as percent of GAPDH for three independent experiments with qRT-PCR in duplicate. Bars = SD. (**C**) Western blot: RAB25 protein level is decreased by ZEB1 overexpression during 1 to 5 days of DOX treatment in H358 FlipIn ZEB1cells. E-cadherin is decreased as well. Actin is the loading control. Protein molecular weights are indicated in kDa on the left.

From our previous identification of ZEB1-correlated genes in lung cancer using Affymetrix arrays, we had confirmed regulatory relationships for *ST14*, *EpCAM*, and *ESRP1* upon ZEB1 gain- and loss-of-function. In addition, *RAB25* expression was negatively correlated with ZEB1 (R = −0.73), similar to that of *E-cadherin* (R = −0.75) [10]. Of interest, EGFR inhibitors are currently used in clinics for non-small cell lung cancer patients with EGFR activating mutations [28,29,30,31] and *RAB25* expression has been reported to correlate positively with EGFR inhibitor sensitivity in NSCLC cell lines [32]. Therefore, we first validated the Affymetrix results by qRT-PCR analyses in 22 NSCLC cell lines and four controls. With the cell lines arranged in increasing *RAB25* mRNA levels (Figure 1A, upper), *RAB25* expression negatively correlated with *ZEB1* expression (Figure 1A, middle), whereas it was positively correlated with expression of the epithelial marker *E-cadherin* (Figure 1A, lower). We confirmed that *RAB25* expression was decreased by doxycycline-induced ZEB1 in H358 NSCLC cells (Figure 1B) and also by TGF-β, a natural inducer of EMT (Figure 1B). RAB25 protein level was decreased by ZEB1 (Figure 1C). Thus, RAB25, an epithelial marker, is repressed during EMT.

**Figure 2:**
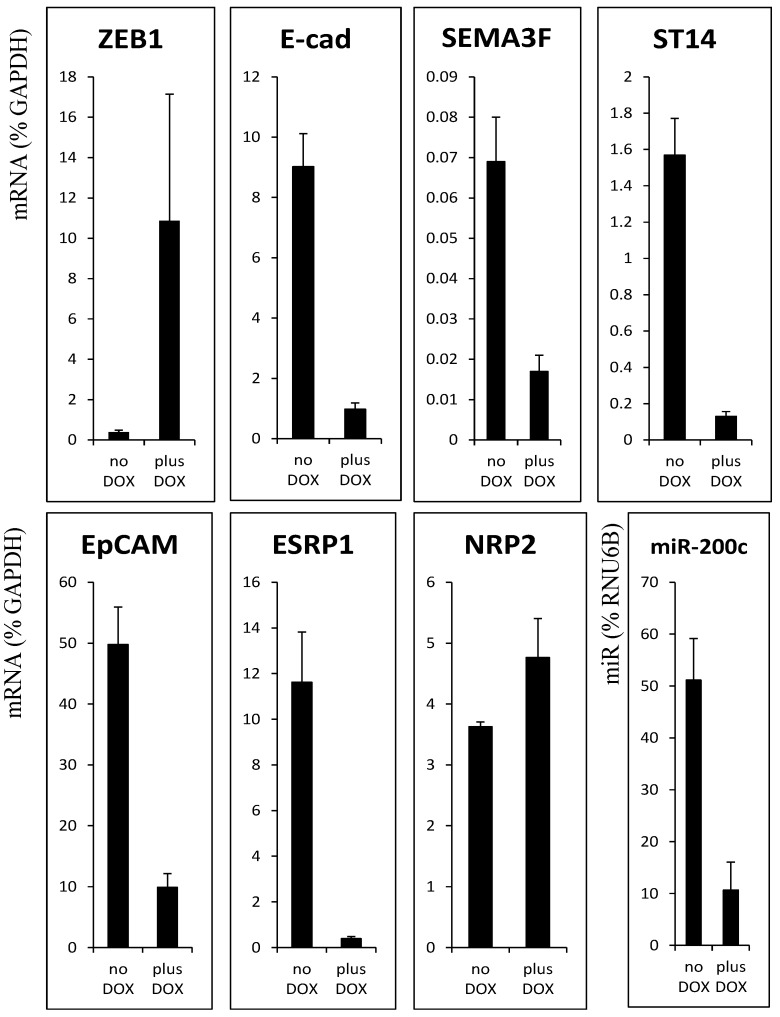
Expression of ZEB1 target gene is decreased in H358 FlipIn ZEB1 cells after 48 h of DOX treatment. mRNA expression was measured by quantitative real-time PCR and values are expressed as percent of GAPDH. For miR-200c, values are expressed as % of RNU6B. Values are the mean from three independent experiments with qRT-PCR in duplicate. Bars = SD.

Next, we asked whether ZEB1 binds directly to the promoters of *RAB25* and the previously identified targets [10]. We first verified decreased mRNA levels of the ZEB1-negatively correlated genes after ZEB1 induction in H358 non-small cell lung cancer cells (Figure 2). To determine whether ZEB1 binds directly to regulatory regions of *ST14*, *EpCAM*, *ESRP1*, and *RAB25*, we identified predicted, evolutionary conserved, ZEB1 binding sites in the promoter regions of these genes, as well as a site in the first intron of *ESRP1* (Supplementary Material).

**Figure 3:**
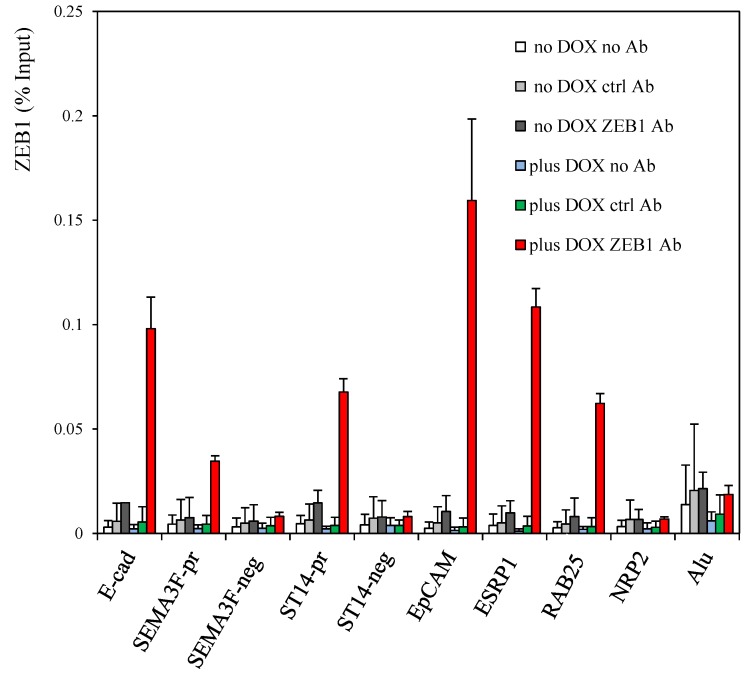
ZEB1 binds the targets genes. ChIP assays were performed with the rabbit anti-ZEB1 antibody on H358 FlipIn ZEB1 cells grown for 48 h in the absence or in the presence of DOX. As negative controls, ChIP assays were performed with IgG from a non-immunized rabbit and also in the absence of antibody. The previously described ZEB1 binding sites in the *E-cadherin* and *SEMA3F* promoters [9] were positive controls. Values are from three independent experiments with qPCR reaction in duplicate and are expressed as % of input. Bars, SD.

We tested ZEB1 binding to these elements by chromatin immunoprecipitation (ChIP) coupled to quantitative real-time PCR analyses with corresponding positive and negative controls (Figure 3). Enriched ZEB1 recruitment was identified for predicted target sites in *ST14*, *EpCAM*, *ESRP1*, and *RAB25*. In contrast, no binding was evident for a conserved E-box sequence in *Neuropilin 2* (*NRP2*), which encodes a common receptor for SEMA3F and VEGF [33], and which seems to be upregulated by ZEB1 predominantly at the post-transcriptional level according to our preliminary data. Also, no enrichment was evident for E-box-negative regions located in *ST14* intron 16 (ST14-neg), *SEMA3F* intron 13 (SEMA3F-neg) and *Alu* sequences [9] (Supplementary Material). Together, these results demonstrate that ZEB1 binds *ST14*, *EpCAM*, *ESRP1*, and *RAB25* when these genes are silenced following ZEB1 induction. 

### 3.2. Decrease of Histone Acetylation by ZEB1 on Target Genes

**Figure 4:**
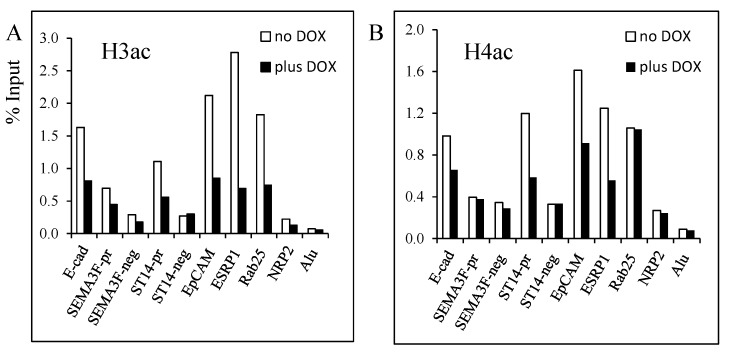
Decrease of histone H3 and H4 acetylation induced by ZEB1 binding on target genes. ChIP assays were performed with the rabbit anti-acetyl H3 (**A**), and anti-acetyl H4 (**B**) antibodies on H358 FlipIn ZEB1 cells grown for 48 h in the absence (white bars) or in the presence (black bars) of DOX. Results are expressed as % of input. Two independent experiments were performed with qPCR reaction in duplicate and one representative experiment is shown.

Next, we focused our study on histone acetylation because ZEB1, as a repressor, recruits class I and II histone deacetylases (HDACs) in pancreatic tumors and nicotinamide adenine dinucleotide (NAD)-dependent HDAC SIRT1 in prostate cancers, while in colon cancer cells, ZEB1 recruits BRG1 to repress *E-cadherin*. To our knowledge, the mechanism of ZEB1 repression in lung cancer cells during EMT is not known. In our previous study [9], we found that *SEMA3F* was repressed by ZEB1 and that SAHA, a HDAC inhibitor, reduced ZEB1 binding. This result suggested that histone acetylation was modified upon ZEB1 binding. In the present study, we checked this hypothesis for *SEMA3F* as well as the new ZEB1 targets that we identified. Using ChIP assays, a decrease in histone H3 acetylation was found at each of the tested target genes (Figure 4A). Similarly, with the exception of *RAB25*, decreased histone H4 acetylation was also noticed (Figure 4B). 

**Figure 5:**
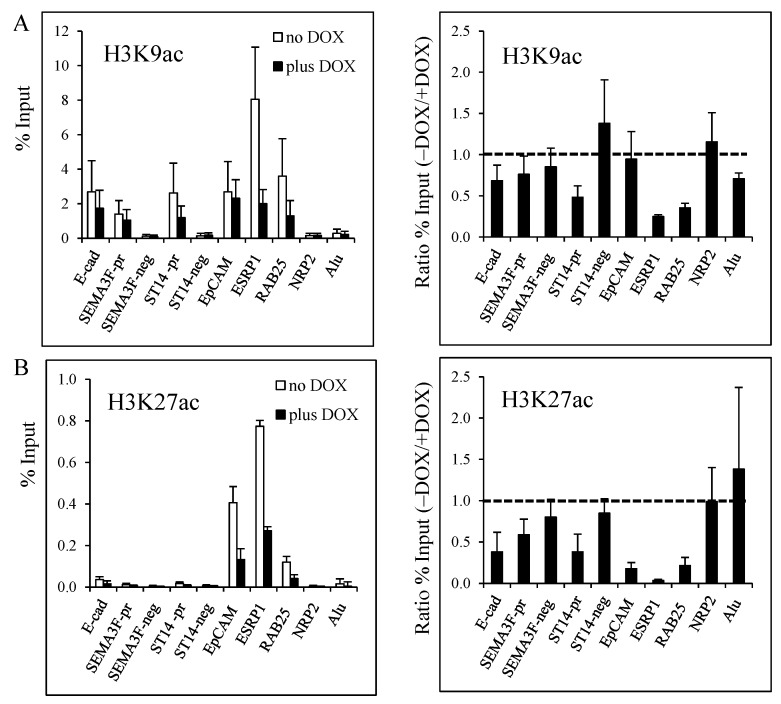
Decrease of histone H3K9 and H3K27 acetylation induced by ZEB1 binding on target genes. ChIP assays were performed with the rabbit anti-acetyl H3K9 (**A**), and anti-acetyl H3K27 (**B**) antibodies on H358 FlipIn ZEB1 cells grown for 48 h in the absence (white bars) or in the presence (black bars) of DOX. Results are expressed as % of input (left column). The ratio of the % input obtained without and with DOX for each experiment is shown in the right column with a value 1.0 indicating no change. Values are from three independent experiments with PCR in duplicate. Bars, SD.

Next, we examined specific histone H3 lysine residues for acetylation. Decreased H3K9 acetylation was found for *ST14*, *ESPRP1* and *RAB25*, but not for *EpCAM* (Figure 5A). Loss of H3K27 acetylation was identified for all target genes, with *EpCAM*, *ESRP1*, and *RAB25* being the most responsive (Figure 5B). Even though the *E-cadherin*, *SEMA3F*, and *ST14* promoter sites had relatively little H3K27 acetylation at baseline, ZEB1 induction caused a further decrease.

In summary, ZEB1 binding to its target gene sequences induces a decrease in histone H3 (H3K9, H3K27) and H4 acetylation, with decreased histone H3 acetylation, especially H3K27, being the most consistent change.

### 3.3. Decrease of Histone H3K27 Acetylation by ZEB1

**Figure 6:**
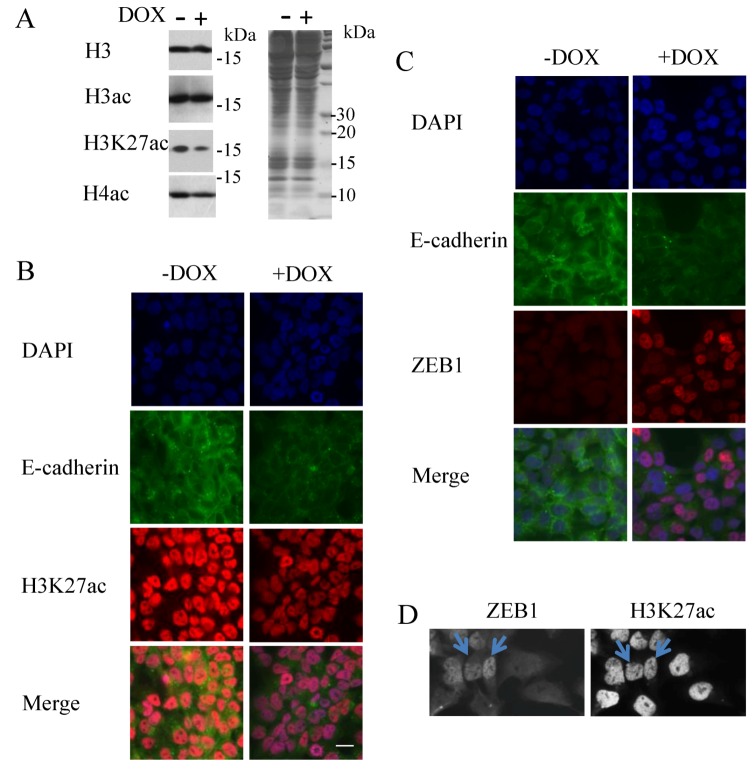
Histone H3K27 acetylation: a mark of ZEB1 induction. H358 FlipIn ZEB1cells were grown for 48 h in the absence (−) or in the presence (+) of DOX. (**A**) Western blot for H3, H3 acetylation, H3K27 acetylation, and H4 acetylation. H3 staining is the loading control. Protein molecular weights are indicated in kDa. This blot is representative of three independent experiments (left panel). Protein staining with Coomassie blue is on the right. (**B**) Immunocytochemistry for H3K27 acetylation (red) in H358 FlipIn ZEB1cells with E-cadherin (green) as a read-out for ZEB1 induction. E-cadherin staining was performed with the mouse anti-E-cadherin. Blue color indicates DAPI nuclear staining. One image is reported and representative of two independent experiments with three pictures taken for each well. Bar scale: 40 µM. (**C**,**D**) E-cadherin, detected with the mouse anti-E-cadherin-antibody, is a read-out of ZEB1 expression: (**C**) Co-immunostaining for *E-cadherin* and ZEB1(detected with the rabbit ZEB1-antibody) in H358 FlipIn ZEB1 cells without and with DOX. E-cadherin staining was decreased when ZEB1 was induced and detected with the rabbit antibody. (**D**) Additional control for co-staining of ZEB1 and H3K27ac. H3K27 acetylation (detected with the rabbit H3K27ac-antibody) was performed with ZEB1 stained with the mouse antibody in ZEB1-induced H358 cells. H3K27 acetylation staining is less intense in positive ZEB1 stained cells as indicated by arrows. Six pictures were taken per well and one representative image is shown.

To determine whether ZEB1 induces changes in histone acetylation at a global level, we examined H358 cell lysates by western blot upon ZEB1 induction. Compared to controls, global H3K27 acetylation was decreased upon ZEB1 expression, whereas no global decrease was evident for total H3; total H4 acetylation was minimally affected at best (Figure 6A). By immunocytochemistry, we also found that H3 and H4 acetylation staining was unchanged (data not shown), whereas H3K27 acetylation staining was decreased (Figure 6B). In order to maintain use of the same antibodies for both immunocytochemistry and ChIP assays, we used E-cadherin staining as a marker of ZEB1 induction. We verified decreased E-cadherin staining when ZEB1 was induced (Figure 6C). Even with the use of the mouse anti-ZEB1 antibody, which had a low sensitivity in our hands, we could see that the ZEB1 expressing cells were less positively stained for H3K27 acetylation (Figure 6D). These results suggest that H3K27 loss of acetylation is a mark of ZEB1 induction.

### 3.4. Modulation of Histone H3K27 Methylation by ZEB1

**Figure 7:**
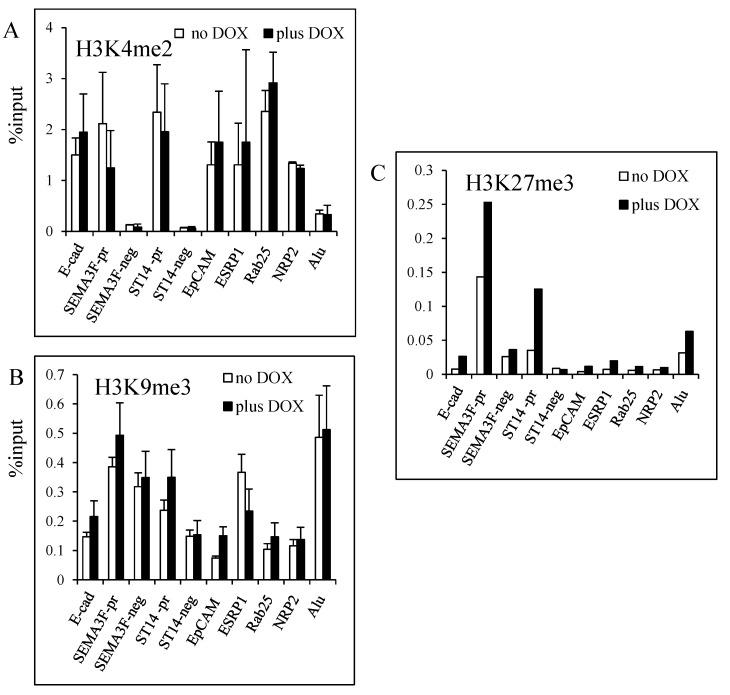
Histone H3K4, H3K9 and H3K27 methylation upon ZEB1 induction. ChIP assays were performed with the rabbit anti-H3K4me2 (**A**), anti-H3K9me3 (**B**) and anti-H3K27me3 (**C**) antibodies on H358 FlipIn ZEB1 cells grown for 48 h in the absence (white bars) or in the presence (black bars) of DOX. Results are expressed as % of input. (**A**,**B**) Values are from three independent experiments with PCR in duplicate. Bars, SD. (**C**) values are the average of two experiments.

Since HDACs interact with many histone methyltransferases, we performed ChIP experiments for H3K4me2, H3K9me3, and H3K27me3 (Figure 7). H3K4 dimethylation (a mark of active chromatin) did not change substantially (Figure 7A) and either no change or a tendency to increased H3K9 trimethylation (a mark of repression on gene promoter) was noticed when ZEB1 was induced (Figure 7B). *Alu* sequences were trimethylated on H3K9 as expected. However, a more consistent enrichment for trimethylation of H3K27, a mark of gene repression, was obtained for the target genes. Of note, the initial H3K27me3 levels without ZEB1 induction were different for the tested genes. *SEMA3F* and *ST14* promoters had the highest level of K3K27 trimethylation (Figure 7C) that could reflect their low expression (Figure 2). These results indicate that ZEB1 binding to its target sequences affects H3K27 with some enrichment for tri-methylation.

### 3.5. DNA Demethylation Agent and Histone Deacetylase Inhibitor Increase ZEB1 Target Gene Expression

**Figure 8:**
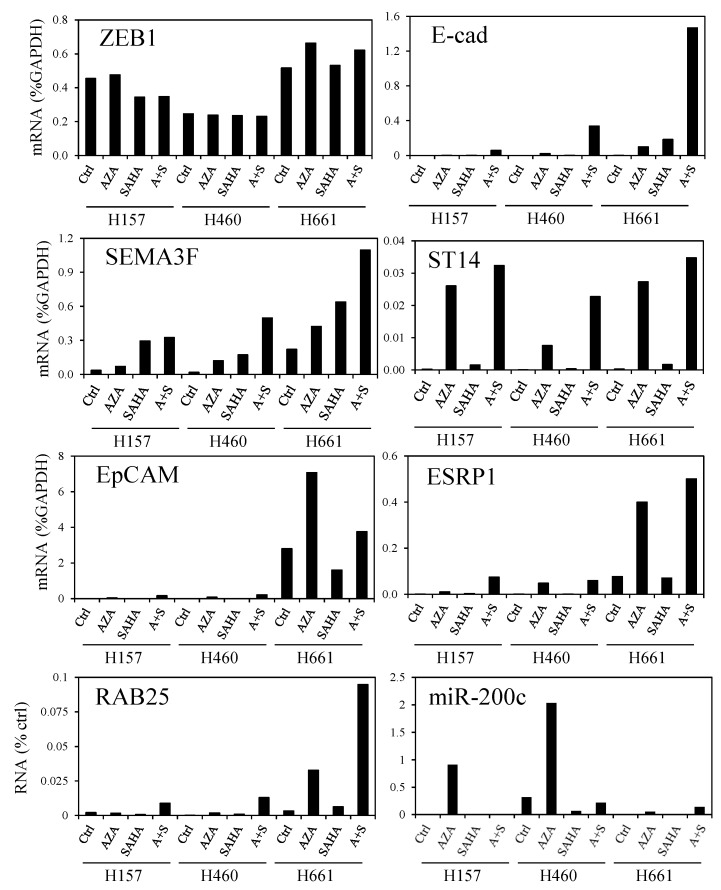
DNA demethylation and histone deacetylase inhibition increase ZEB1 target gene expression. H157, H460, and H661 NSCLC cell lines were treated by AZA, SAHA, and the combination (A + S). Gene expression was measured by qRT-PCR. For mRNA expression, values are expressed as percent of GAPDH, and for miR-200c they are expressed as % of RNU6B. Two independent experiments were performed with qPCR reaction in duplicate and one representative experiment is shown.

Since loss of histone acetylation seems to be a mark of ZEB1 induction, we treated three NSCLC cell lines with vorinostat (SAHA), a HDAC inhibitor, either alone or in combination with 5-aza-2'-deoxycytidine (AZA), an inhibitor of DNA methylation. This strategy was chosen because DNA methylation is abnormal in cancer cells [34] and because combination of DNA methylation and HDAC inhibitors has efficacy in patients with advanced non-small cell lung cancer [35]. The three cell lines (NCI-H157, NCI-H460, NCI-H661; hereafter H157, H460, H661) have a mesenchymal phenotype compared to H358 cells and express ZEB1 endogenously [10]. The SAHA and AZA treatments partially restored mRNA expression of the ZEB1 target genes but the response was cell line-dependent, with H661 cells being the most responsive (Figure 8). The response was also gene specific. Indeed, *SEMA3F* expression was responsive to SAHA alone, while *E-cadherin* and *RAB25* mRNA levels were most stimulated by SAHA and AZA. The other ZEB1 target genes, *ST14*, *EpCAM*, and *ESRP1*, responded significantly to AZA alone.

Lastly, since EMT is under the control of micro-RNAs of the miR-200 family, we examined the expression of miR-200c after SAHA and/or AZA treatment. AZA treatment was efficient by itself to restore miR-200c expression in H157, H460, and H661 cells (Figure 8). However, the induced miR-200c level was less than the endogenous level observed in H358 cells (Figure 2). Of note, ZEB1 mRNA levels were not affected by AZA/SAHA treatment in these cell lines. 

Together, these results suggest that combined treatments targeting epigenetic mechanisms would reduce EMT.

### 3.6. Histone H3K27 Acetylation in Lung Cancers

**Figure 9:**
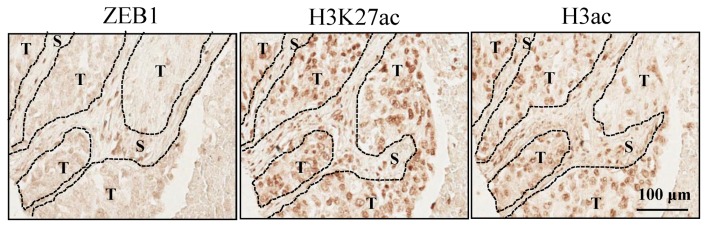
Staining for ZEB1, H3K27 acetylation, and H3 acetylation on a TMA. One example of a lung squamous adenocarcinoma is shown. T and S indicate tumor and stroma compartments respectively.

To determine whether a correlation exists in human tumors between ZEB1 expression and H3K27 acetylation, we examined corresponding immunohistochemical stainings on a commercial tissue microarray containing a series of human lung cancers. Because of sample quality, some tumors were excluded leaving, for analysis, 43 adenocarcinomas, 36 squamous cell carcinomas and a group of 20 other lung tumors (called “others”) with a limited number of adenosquamous carcinomas, atypical carcinoid large cell carcinomas, and papillary adenocarcinomas. 63% of the tumors were positive for ZEB1 with a nuclear staining confirmed to elongated cells in the stroma compartment. Among these tumors, 31% were also ZEB1 positive in the tumor compartment (Figure 9). These results are consistent with ZEB1 being predominantly expressed in the stroma. Histology, tumor grade, and metastatic lymph nodes did not affect ZEB1 scores.

In contrast, every tumor was positive for H3 acetylation and H3K27 acetylation in both compartments (Figure 9). Interestingly, H3K27 acetylation scores were statistically higher in the tumor compartment than in the corresponding stroma for all pathological samples with a median ratio of 1.06 (*p* < 0.0001). This difference was confirmed with histology (adenocarcinomas, *p* = 0.0423; squamous cell carcinomas, *p* = 0.0004; and others, *p* = 0.0177), tumor grade, and metastatic lymph nodes. Similar statistically significant results were found for H3 acetylation for all pathological samples (*p* < 0.0001), and confirmed for squamous adenocarcinomas (*p* = 0.0257) and others (*p* = 0.0022), but not for adenocarcinomas (*p* = 0.0663). These results suggest that H3K27 acetylation is preferentially higher in the tumor compartment, whereas ZEB1 is preferentially expressed in the stroma of the tumor. 

## 4. Discussion

Emerging data provide evidence for the role of EMT in lung cancers [36], including the description of circulating lung cancer cells with an epithelial-mesenchymal phenotype [37]. Although E-cadherin loss is a classic feature of EMT, it has been considered as a late event in tumor progression. The use of additional markers has demonstrated that a majority of NSCLCs have EMT features. Previously, we and others found that ZEB1 was predominantly responsible for the loss of E-cadherin in lung cancer cell lines [7,8]. More recently, using transcriptomic data from 38 NSCLC cell lines, we identified genes most correlated with ZEB1 expression [10]. For a subset of these genes, we confirmed that their expression was responsive to both overexpression and knockdown of ZEB1, but did not determine whether the changes were the direct consequence of ZEB1 binding. In the present study, this is now demonstrated for *ST14*, *EpCAM*, *ESRP1* and, in addition, for *RAB25*.

*RAB25*, an epithelial-specific member of the Rab family of small GTPases, can act both as a tumor promoter and suppressor. When highly expressed in ovarian cancer cells, RAB25 facilitates invasion [38,39]. It is also pro-tumorigenic in human colon cells [13]. Conversely, loss of RAB25 is associated with a poor prognosis in ER-negative breast cancer, and RAB25 behaves as a tumor suppressor in breast cancer cell lines by decreasing cell migration/invasion and affecting pathways including the VEGF-A/VEGFR-1 autocrine loop [40]. Some of these discrepancies could be due to CLIC3, which is involved in α5β1 integrin trafficking necessary for cell invasion [12]. In the absence of CLIC3, RAB25 acts as a tumor suppressor, whereas in the presence of CLIC3, RAB25 increases tumor aggressiveness [12]. RAB25 is involved in intracellular vesicle trafficking in the regulation of epithelial polarity and transformation. Changes in the localization of integrin β1, which affects tumor cell invasion/migration, have been associated with both increased and decreased RAB25 [13,38]. RAB25 expression has also been reported to affect Ras signaling, the recycling of EGF or TGF-β receptors, and was found to bind Smad4 and TGF-βR1 [14,15], suggesting that RAB25 plays an important role in the EMT phenotype.

Interfering with EMT in cancer requires detailed knowledge of how target genes are affected, including the epigenetic modifications of histones. In H358 NSCLC cells, ZEB1 binding resulted in decreased acetylation of histones H3 and H4, especially a decreased acetylation on residues H3K9 and H3K27 on target genes. By global analyses (Western blot and immunocytochemistry), although ZEB1 induction in H358 cells did not lead to detectable decrease in H3 or H4 acetylation, we did identify a global decrease in H3K27 acetylation. Thus, decreased H3K27 acetylation may be a mark for ZEB1-positive cells undergoing EMT.

Decrease in lysine acetylation on ZEB1 target genes is in accordance with the mechanism of transcriptional repression induced by ZEB1. Indeed, ZEB1 can recruit class I HDACs, HDAC1 and HDAC2 [18,20,41], and the nicotinamide adenine dinucleotide-dependent HDAC SIRT1 for *E-cadherin* repression [21]. Indeed, increased H3K9 acetylation (a mark of active chromatin) and increased RNA pol II occupancy were found on the *E-cadherin* promoter concomitant with *ZEB1* knockdown in DU145 prostate cancer cells. As might be expected, these changes were associated with increased *E-cadherin* expression [21]. Of note, Sirtuins also act as mono-ADP-ribosyltransferase (for review: [42]) and this function suggests that mono-ADP-ribosylation might be affected by ZEB1 binding as well.

Treatment with SAHA (a HDAC inhibitor), either alone or in combination with AZA (an inhibitor of DNA methylation), partially restored epithelial gene expression in three NSCLC cell lines that exhibit a more mesenchymal phenotype than H358 cells. The response was cell line-dependent and gene-specific. SAHA was mostly effective to restore *SEMA3F* expression but had little effect on other target genes by itself. One explanation could be that on the non-responsive genes, ZEB1 associates with Sirtuins, members of class III HDACs that are insensitive to SAHA. This would explain the lack of response of H157 cells for *E-cadherin* expression with SAHA and other class I and II HDAC inhibitors [43]. AZA was more efficient to restore ZEB1 target gene expression. It could be explained, in part, by the induction of miR-200c expression that is often silenced by DNA methylation in NSCLC cell lines [44,45]. miR-200c is a member of the miR-200 family that negatively regulates EMT and affects a multitude of extracellular matrix and cell adhesion molecules [17,46], and its loss is associated with an aggressive, invasive and chemoresistant phenotype in NSCLC [44]. Further experiments would be necessary to explore histone acetylation and DNA methylation on ZEB1 target genes.

ZEB1 mediates repression, by interacting with the corepressor CtBP [19] which associates with HDACs and several other partners including the Polycomb complex PRC2 which methylates H3K27. ZEB1 also interacts with the histone methyltransferases G9a (EuHMT2) that mono-methylates H3K27 [47], the co-repressor CoREST, and the histone demethylase LSD1 (for reviews see [22,23]). Indeed, when H3K27 acetylation (a mark of transcriptional activation) is lost, G9a could first mono-methylate H3K27 which could be further di- and tri-methylated by the Polycomb complex PRC2, establishing a mark of stable transcriptional repression [48]. Interestingly, within the same region, H3K27 methylation can be found with H3K4 methylation, a mark of active promoter. The presence of both marks defines bivalent domains within regions enriched for genes poised for activation in pluripotent cells [49]. Our preliminary results show that ZEB1 increased trimethylation of H3K27 on selected target genes and that H3K4me2 did not change drastically upon ZEB1 binding. They suggest that ZEB1 would recruit PRC2 and would create bivalent domains during EMT. Further experiments, beyond the scope of this study, would be necessary to explore this issue.

In human NSCLC tumors, we restricted our analysis to ZEB1 staining and H3K27 acetylation because loss of H3K27 acetylation is critical for transcriptional repression. Although H3K27 acetylation is found in a large proportion of cells either in the tumor or the stroma compartment, H3K27 acetylation scores were higher in the tumor compartment. In contrast, ZEB1 was more often expressed in a restricted number of cells in the stroma of the tumor. This result suggests that there is a reciprocal relationship between H3K27 acetylation and ZEB1 expression in patient tumor samples. The nature of the ZEB1 positive stroma cells is still unknown. The possibility that these cells represent tumor cells that have undergone EMT is supported by Mink *et al.* [50] and our previous work [10]. In that case, decreased H3K27 acetylation might mark a region where tumor cells have escaped the primary tumor in the metastatic process.

Changes in histone modifications have been described in NSCLC tumors, without focusing on EMT. Gain of H4K5 and H4K8 acetylation, loss of H4K12 and H4K16 acetylation and H4K20 trimethylation were described (for reviews see [51,52]). Levels of H3K4 dimethylation and H3K18 acetylation have also been reported as independent predictors of clinical outcome in adenocarcinomas of different origins (for reviews see [52,53,54]). High level of SIRT1 was found in lung cancer [55] and increased histone methyltransferase G9a, which methylates H3K9 and mono-methylates H3K27 [47], was correlated with poor prognosis [56]. Indeed, G9a was reported to be responsible for EpCAM repression with an enrichment for histone H3K9 dimethylation and promoter methylation [56]. Deregulation of histone methyltransferases was reported for oncogenic transformation of human bronchioepithelial cells [57] and mutations of the histone methyltransferase, SETD2, were recently identified by large scale DNA sequencing of lung adenocarcinomas [58,59]. It is possible that some of these epigenetic modifications might be specifically linked to the development of EMT features.

## 5. Conclusions

In summary, we found that ZEB1 binds directly to the target genes we identified: *EpCAM*, *ESRP1*, and *ST14*. We identified *RAB25* as a new ZEB1 target gene. ZEB1 binding was associated with reduced histone H3 and H4 acetylation and some increased methylation of H3K27. Decreased H3K27 acetylation could be detected by global analysis. Our results represent a step towards identifying the chain of events leading to changes in gene expression mediated by ZEB1 during the EMT process in lung cancer. An effective therapeutic intervention will require more detailed knowledge and the availability of a wider repertoire of agents, such as those that inhibit EZH2 and other methyl transferases [60] in addition to DNA methylation and HDAC inhibitors recently described for their efficacy at low doses [35].

## Supplementary Files

Supplementary File 1 Supplement Material (PDF, 131 KB)
