# Erratum: Roche, J. et al. Global Decrease of Histone H3K27 Acetylation in ZEB1-Induced Epithelial to Mesenchymal Transition in Lung Cancer Cells. *Cancers*, 2013, 5, 334–356

**DOI:** 10.3390/cancers8120114

**Published:** 2016-12-15

**Authors:** Joëlle Roche, Patrick Nasarre, Robert Gemmill, Aleksander Baldys, Julien Pontis, Christopher Korch, Joëlle Guilhot, Slimane Ait-Si-Ali, Harry Drabkin

**Affiliations:** 1 Department of Medicine, Hematology Oncology Division, MUSC, 96 Jonathan Lucas St., Charleston, SC 29425, USA; nasarre@musc.edu (P.N.); gemmill@musc.edu (R.G.); drabkin@musc.edu (H.D.); 2 CNRS FRE 3511, University of Poitiers, 1 rue Georges Bonnet, F-86022 Poitiers Cédex, France; 3 Department of Medicine, Nephrology Division, MUSC, Ralph H. Johnson Veterans Affairs Medical Center, Charleston, SC 29425, USA; aleksander.baldys@siemens.com; 4 Epigénétique & Destin Cellulaire, CNRS UMR 7216, University of Paris Diderot, Sorbonne Paris Cité, F-75013 Paris, France; julien.pontis@univ-paris-diderot.fr (J.P.); slimane.aitsiali@univ-paris-diderot.fr (S.A.-S-A.); 5 CU DNA Sequencing and Analysis Core, University of Colorado, School of Medicine, Anschutz Medical Campus, 12801 E. 17th Ave., Aurora, CO 80045, USA; Christopher.Korch@ucdenver.edu; 6 INSERM, CIC 0802, CHU de Poitiers, F-86021 France; joelle.guilhot-gaudeffroy@chu-poitiers.fr

The authors wish to make the following correction to their paper [1]. The sequences of actin primers used in qRT-PCR presented in Table 1 are wrong. The corrected sequences are:

Actin forward primer: 5′ ACCGCGAGAAGATGACCCAG 3′
Actin reverse primer: 5′ AGGTCCAGACGCAGGATGG 3′

The authors would like to apologize for any inconvenience caused. The change does not affect the scientific results. The manuscript will be updated and the original will remain online on the article webpage.

## References

[1] Roche J., Nasarre P., Gemmill R., Baldys A., Pontis J., Korch C., Guilhot J., Ait-Si-Ali S., Drabkin H. (2013). Global decrease of histone H3K27 acetylation in ZEB1-induced epithelial to mesenchymal transition in lung cancer cells. Cancers.

