# Supplementary material for: Global Decrease of Histone H3K27 Acetylation in ZEB1-Induced Epithelial to Mesenchymal Transition in Lung Cancer Cells
Source: Cancers (Basel). 2013 Apr 3;5(2):334–56. doi: 10.3390/cancers5020334 (PMC3730320; doi:10.3390/cancers5020334)
Supplement: Supplementary File 1 — Supplement Material (PDF, 131 KB) [file cancers-05-00334-s001.pdf]

Supplement Material

Localization of E-box and primers used for ChIP qPCR: Red: 5'UTR, Blue: Exon 1, Green: ATG initiator codon, Yellow: conserved E-box predicted by Mulan with consensus sequence in bold, Italics: additional E-box, Underlined: primers for qPCR.

EpCAM

E box: conservation in 2 species (human, macaque)
ccTGGGAA**CACCT**TTTCTTACATCTTCAAGTGCTAGAAATGCTTATGAAAACGAAAAAAG
AATTATTAAGAGTAATTATAAAGAAACACTCATTTTCTTCCCAAGAGAGCCAAGAtttcttcttttc
ctcttctttcttttttttttctttCTAATTTCAAAGGAGTATAATTAAATTGCCAGGTAAAAGCTCAAAGGTC
TTTTTTATAGTGTTCTGGAAGGTTCTCTGCCTGTGTTTGTATTTCCTTTAGCCTCCACGTTC
CTCTATCCAGTTCCCGCACCCTTCCCCCCAGGCCCCATTCTTCAAGGCTTCAGAGCAGCGC
TCCTCCGGTTAAAAGGAAGTCTCAGCACAGAATCTTCAAACCTCCTCGGAGGCCACCAAA
GATCCCTAACGCCGCCATGGAGACGAAG***CACCTg***gggcggggcggagcggggcgcgcgggcccA***CACCT***
GTGGAGAGGGCCGCGCCCCAACTGCAGCGCCGGGGCTGGGGGAGGGGAGCCTACTCACT
CCCCCAACTCCCGGGCGGTGACTCATCAACGAGCACCAGCGGCCAGAGGTGAGCAGTCCC
GGGAAGGGGCCGAGAGGCGGGGCCGCCAGGTCGGG**CAGGTG**TGCGCTCCGCCCCGCCG
CGCGCACAGAGCGCTAGTCCTTCGGCGAGCGAGCACCTTCGACGCGGTCCGGGGACCCCC
TCGTCGCTGTCCTCCCGACGCGGACCCGCGTGCCCCAGGCCTCGCGCTGCCCGGCCGGCT
CCTCGTGTCCCACTCCCGGCGCACGCCCTCCCGCGAGTCCCGGGCCCCTCCCGCGCCCCTC
TTCTCGGCGCGCGCGCAGCATGGCGCCCCCGCAGGTCCTCGCGTTCGGGCTTCTGCTTGCC
GCGGCGACGGCGACTTTTGCCGCAGCTCAGGAAGGTGAGGCGCGGATTGGAGCAGAGTT
GTGGAGCTGGGCTGGGCTGGG

ESRP1

E box: conservation in 2 species (human, macaque)
CTCCTCCCCCACCGCTGCCACGTCGTGGTTTGAAGGAGCCAATGGGCCGGCGCCGCCAGG
TGTCTCTTACCTGCACCACGTGGGGGAGGGGGAAGGGGCGGG**CAGGT**AAAGCCACATCC
CAAAACAGAAAAGCTTTCAGCCATTGCGTGCCTCCCGGGGGGGGCAGCCTTGCTCCAGGC
TTTTTGCATAGACGCCCGGGCAACTGAATACAAAAAGGGCAGGCCTCCTGCGCCCTCCTT
CCCACCCCCCTTCCTGCCCTGGGCGTGGAGCACCGAC**CAGGTG**TGGGCTTGGGTGGTTGG
TTACCGCCTTTTGCACTAGCAGTAGCAAGGAAGGGGGGTGGGCGCTCTTTCTTTTTCTCTT
AGAAGAGGGTTTAGCACAGGTTTTTTCGTTCTCACTTCCACACCACCTTACCGCCTCCCGA
CCCCCCCTCTCCCCCTCCCCACCTATCGTCATGACGGCCTCTCCGGATTACTTGGTGGTGC
TTTTTGGGATCACTGCTGGGGCCACCGGGGCCAAGCTAGGCTCGGATGAGAAGGAGTTGA
TCCTGCTGTTCTGGAAAGTCGTGGATCTGGCCAACAAGAAGGTATTTCTCCACATTTTCGT
CTAAATGCAAGGAATGGGGCAAGAAGTTTGTGGTAGAGGTTTGGGCGGGGAGGGGGGTG
GAGGTAAGTAAATAAGTGCTCTTGTTTGCCCACTTGTGAGTCTGGACCCGAGGCCTAGGG
AAGCTAGAGTAAAACCAAACTTATTGGCCAACTGCCTTGGAGCCATTTCAGTCCTCCGCA
ACTTAGCT**CAGGTG**GAGAGGCCGGCGCTACCGAGCCGGGTTCTTTGCCCGTCGGGGGAC
GCTCCGCCGAGACGAGGTGGGGTCTCGGAGGCCCCGGGGTCCACTTCCAGGGCTTTTCCG

ACCTATCGGGTGGGGGGTGGGGCGGGGGGCAGGAACGCACCGGAACCGATGGCGAGTCG
AGAGCTTTGATTCTGCGTCCGGACCCCAAGAGAGGCGCGG

RAB25

E box: conservation in 4 species (human, macaque, dog, mouse)
AGTTGGGGGACCGGCCCTCACCACAGATTCCAGAAGCCCAGCAATGCACACTCAGCCCTC
AGTGGGCTGTCTCTGAAGGTCCTGTCCCTTTTTCGCTTCCCCCCCGCTGGAGCTGCTTCTCC
CGCTTGCGGGAGCCCAGGCTGAGAGCAGACACCCAACCTGTCGA**ACCTG**TCTGACGTCAT
CATCTCTCCACC**CACCTG**GGCCCCAGGTCTCCAGCCACCCCGCTCTTCCTGTTCTCAGCTT
CCGTCCTCTCTGCTTCCTTACAGCACCCCCACCTGCCAGAGCTGATCCTCCCTAGGCCCTG
CCTAACCTTGAGTTGGCCCCCAATCCCTCTGGCTGCAGAAGTCCCCTTACCCCCAATGAGA
GGAGGGGCAGGACCAGATCTTTGAGAGCTGAGGGTTGAGGGCATTGAGCCAACACACA
GATTTGTCGCCTCTGTCCCCGAAGACACCTGCACCCTCCATGCGGAGCCAAGATGGGGAA
TGGAACTGAGGAAGATTATAACTTTGTCTTCAAGGGTGAGTTGGGTTCCCTGAAGCAAGA
GGAAGCCTGAGAGACCCAGAAAGAGATTGAATAGAGCCAGAGAGGGCCCCCTCCTGTTT
TTTTATCTCTTACCAAGACCTCCTGGGGCTCCTCATTCCTTCAGAAAGAAAGCTGCAGGGA
GTGGGGGAGGGTCTGGTTGCAGGGGCAAAAGTCAGGGAGCTTCTGAGTATGCTCTACTGT
GGTAAATAATGATGTTGATTCCTCCTTAAGAGTGCTGAGTTAGGCTCCTCTGCCCCGTTCC
TAATAGAAACCCAAATCCCTGGTGTCAGCCAGTGCAGCACAAGAGATGGAGGTCAGACTT
CAGAA

ST14-pr

E box: conservation in 2 species (human, macaque)
Human (hg18) chr11:129534300-129535100
GGAATCCAGGGAAAGGGGGCATTTGAGGAAGCATCAGATGCTGCAAGTGGGCGAGGGGC
GTTGGGATGGCTCAGAGGGCATCTGGTGGCACAGCTCATGTCACTGGGCCTCCCCAGGGT
AGCCGGGCGCAGGCCAGCAAGCGCCTCCCGCCTGGAGAACAGGAGGCTGCGTAGCCGAC
GGGGAGCCTGGGCCAGGGGCGGTTCTGGTTTCTGTTTGGTTTTGGGGGGTTCATAAATGC
GATTTAGTCCTGTAGCAAAGTGAGCAAGGTGAAGGGGAGAGACCGGAGGCGCATGAGGG
AGGAGGGAAAAGATGGTTGGCATCAAGGAGGTGGATAAAGGGCTGCCCTCCCGGGGCAG
GGCCCCTGGAGGGCCGGAGGGGTGGTGAACGCCGTCGTGGTGATGGTGAGGGCCTTAGC
GACCACCACCCGAGTGAGGCCCCCTCACCTTCAGGCCCAGAGCCGAGGGGAGGAGCCTC
CCCCAgggccggcgggggcgggcaggtgagccgggcaggtgcgcaggcgagcgcggcgggctgggcgggcCGAGGCCACA
CCCTGAAACTACCTGCGTGGGCCGGGCCGGAGTGTGAGAGCGGAGCTGCAGCCGGAGAA
AGAGGAAGAGGGAGAGAGAGCGCGCCAGGGCGAGGGCACCGCCGCCGGTCGGGCGCGC
TGGGCCTGCCCGGAATcccgccgcctgcgccccgcgccccgcgccctgcgggccATGGAGCCGGCCGCCGG
CAGGGACGACGCCTGTGAGACCCGCGAGCGGCCTCGGGGACCATGGGGAGCGATCGGG

ST14-neg

Human (hg18) chr11:129583242-129590000
CAGGTTCGTAAGATAACTAATTTTTGGTCATCAGATTAAAAATGTATTCAAGGAAGGGGG
GGTAAAGAGTTCAAGAGGGATTTCATACTGCAGCTGGAGGCACTTAGTAGTTTCTAGCTT
AGGGTCAAGACCAAGTAAGAGCCGGCCCCATCGTCTTCTCGTAGCAGCAGCTGTGCCTCA
TCCATGCGGAATCCTCTGGGAACGCGCGGGGCCGCGAGGCCGTGTGCACAGACCCGAGT
GACCGCGCAGTCTCATAGCGGCTCTCCCTACCAGGTACTCAGACCCCACGCAGTGGACGG
CCTTCCTGGGCTTGCACGACCAGAGCCAGCGCAGCGCCCCTGGGGTGCAGGAGCGCAGGC
TCAAGCGCATCATCTCCCACCCCTTCTTCAATGACTTCACCTTCGACTATGACATCGCGCT
GCTGGAGCTGGAGAAACCGGCAGAGTACAGCTCCATGGTGCGGCCCATCTGCCTGCCGGA
CGCCTCCCATGTCTTCCCTGCCGGCAAGGCCATCTGGGTCACGGGCTGGGGACACACCCA
GTATGGAGGTAAGCTTCGGGCTGACCTAGGGCTCCGCAGAGGGCCTGGGGCCTTTCTCCA
AGGCCGTGTTTCCTCTGCGTGTCCGGTCTCGGGGCGGGGGGCTGCTCCAGTCTCCCTCTGC
CTGGCAGAATGAGGCTGTCCAGGGCGGAATGGAGGCCTTTagtaaaaggggcatgggatttgcagcctggg
ctcaggattgaaggttggctttgcccctaactagctgagtgaccttgagcgagttgcttaagctctgatcctgtttgtgcatgcgcgtaatgaagatagc
aacacctTAAGCCCAGTGGTGCTTAAGGCACGTGAGGGGGTGGGTATGAAGGGAAGCAGGCC
GTTAGTTGGCAAATGCTAGATTGAAAGAATCCACTGTACAGCATATGCTGAACATTTGTC
GTCTTTGGAGAGGAAAGAAAACGAACAGGACTTACAGCTCCAATGCCCTCACAGAACCC
CGCGGGCATTTGCGGCGCCTTCCCTCCGCCTCTCTGC

NRP2

E box: conservation in 4 species (human, macaque, dog, mouse)
AAACATTAAAGCAGTAGCTAGGCGCTTTAATGAAGTTAGCCTGCTGAAACCAGGGGAGGT
ATGCCCGAAGTACACCTGAAAGCACTTTGATTATCGCATCGGCTTCAAAAGAAGCCCCTT
CCTTCGTTTTTTAGCTCTGCTGCCCTGAGAAATAGAATTACACCTTTATATTCAGGTTAATT
TTGCTGTCTTGCTGAGGCCTGAAAGCATTTCTAAGCACTCTGAAGATGTAAGGGAAGCGG
GCTGTTCCTCTCCATCCTGCCCGCCACGTCGCAAGCCCAAATGGACAGAGGGTGCTCAGA
ACCCTTCAGTTTGAAGCATGTCCTAAGAATGTAAAGTCCAGCACAATAAATGCCTTCCAG
TTCTGAAAAAGATAACTTCCTACTTTCTGCTAACATTGAGTCAATCGGGTTCCACAGGTTT
GCTTTaaaaaaaaacaaatcaaaccaaaacaaaacaaaacCTCAGATAATCCCTGGCCTTCATAAGCTATCCCT
CCCCTAACACACACGCTGCCTTTAGCTTTTTAATATCATCACCTCGCTCTCCAtttccttttgttttata
atgcttgtgtttctttctttcttcgtcttcctctttttccatttttctctttggctggtctctctgcttagcattcctttcatttccttttgcatctctcttcctctaggattt
tagttcctcgtttctgcttgttgtttccttttctGGTCACAGCTGCCCCCTAATGGTTTTCAAGATCATTACTGCTT
TGGACTAGAGGAAAGAGGGAGGCAAAGAGCTAGAGAAGGAAAGTTAGAGAGAAACATA
AAATGTGTCTTTGCTTTTAACTTTGCCATATCGTCTCAATATCTCTGGAAGAATATGCAAA
ACCTGCATCAATGGGGAAGAAGTGAACTGGAAAACTAAGGGA***CAGGTG***TGAAGTTTGTA
CAGGATACTTTTTCGTGTAATTTGAATTTTTAAAAGATGTGTACTACttattttctttttatttaattttgaaat
aaataaataGTATCTGAGTCTGGAGACTGAAAAAGAGACTGCCTCTATAAATGATTTCTTGGTT
GCAGCAGGAAAGCAAGCGCCATGCCAGCCCCCTCCCCATCAGTGTGCTCATCCAGATACT
AAGGGTTGATTGGCAGCTCAGTAAACTATGTAGGAGTAAATATGACGGAGGAGAGTGGG

AACTAATGACTATGGTGGGTGTGAATTAACCCACCCTGGAGTCTCCTCAATAGCAAAAGG
TCAGCTCCCTGACTACTGCCTTTCCATCAAGACTTTCACGCTCAAATTAGCAGCTATTGCC
ATCATAATGTAACAATCACCTGACATGTAGATGTCTAATTAAGGCACCATAAGTCACAGG
TTACAAATTTGAAAACAGCAATAGCAACAATAAAAAAATCCCTTACAAATCTGTGGGGGA
TTTGATAGGGTACATATGCATGTGAATGAAGGCAA
